# *In vivo* and *in vitro* Approaches Reveal Novel Insight Into the Ability of Epicardium-Derived Cells to Create Their Own Extracellular Environment

**DOI:** 10.3389/fcvm.2019.00081

**Published:** 2019-06-19

**Authors:** Noortje A. M. Bax, Sjoerd N. Duim, Boudewijn P. T. Kruithof, Anke M. Smits, Carlijn V. C. Bouten, Marie José Goumans

**Affiliations:** ^1^Department of Cell and Chemical Biology, Leiden University Medical Center, Leiden, Netherlands; ^2^Department of Biomedical Engineering, Eindhoven University of Technology, Eindhoven, Netherlands; ^3^Institute for Complex Molecular Systems, Eindhoven University of Technology, Eindhoven, Netherlands

**Keywords:** epicardium-derived cells, extracellular matrix (ECM), mechanosensitivity, cardiac fibrosis, cardiac remodeling, cardiac repair

## Abstract

Human epicardium-derived cells (hEPDCs) transplanted in the NOD-SCID mouse heart after myocardial infarction (MI) are known to improve cardiac function, most likely orchestrated by paracrine mechanisms that limit adverse remodeling. It is not yet known, however, if hEPDCs contribute to preservation of cardiac function via the secretion of matrix proteins and/or matrix proteases to reduce scar formation. This study describes the ability of hEPDCs to produce human collagen type I after transplantation into the infarct border zone, thereby creating their own extracellular environment. As the *in vivo* environment is too complex to investigate the mechanisms involved, we use an *in vitro* set-up, mimicking biophysical and biochemical cues from the myocardial tissue to unravel hEPDC-induced matrix remodeling. The *in vivo* contribution of hEPDCs to the cardiac extracellular matrix (ECM) was assessed in a historical dataset of the NOD-SCID murine model of experimentally induced MI and cell transplantation. Analysis showed that within 48 h after transplantation, hEPDCs produce human collagen type I. The build-up of the human collagen microenvironment was reversed within 6 weeks. To understand the hEPDCs response to the pathologic cardiac microenvironment, we studied the influence of cyclic straining and/or transforming growth beta (TGFβ) signaling *in vitro*. We revealed that 48 h of cyclic straining induced collagen type I production via the TGFβ/ALK5 signaling pathway. The *in vitro* approach enables further unraveling of the hEPDCs ability to secrete matrix proteins and matrix proteases and the potential to create and remodel the cardiac matrix in response to injury.

## Introduction

Cell-based therapies for the heart aim to recover cardiac function by restoring or preserving contractility of and blood supply in the myocardium. Next to delivery of functional cardiomyocytes as contractile units and restoration of blood vessels for oxygen supply, reconstruction of the cardiac extracellular matrix (ECM) is crucial to eventually rebuild the complex architecture of the myocardium. The cardiac ECM plays a key role in cardiac function, since it provides the structural and functional integrity of the myocardial wall (1). Moreover, the ECM is involved in the presentation of biochemical and biomechanical cues that orchestrate tissue development, differentiation, and homeostasis (2). After a myocardial infarction (MI), the damaged cardiomyocytes are replaced by non-contractile fibrotic scar tissue to prevent rupture of the weakened cardiac wall. Unfortunately, over time, the imbalance between deposition and degradation of the ECM and the disturbance in organization of the ECM will impair cardiac function and ultimately result in heart failure (1, 3, 4). For cardiac regeneration, proper deposition, composition, and organization of the ECM is therefore essential to restore the integrity of the cardiac wall and to improve cardiac function. How cells after transplantation influence the matrix remodeling process in cardiac repair is far from clear.

Recent studies positioned the epicardium, the outer layer of the heart, as a potential source of cardiac stem or progenitor cells for cardiac repair (5–8). The epicardium is essential during cardiac development as it plays a role in the establishment of the myocardium (9, 10). From the epicardial layer, a subset of activated cells undergo epithelial-to-mesenchymal transformation (EMT), and are thereafter defined as epicardium-derived cells (EPDCs) (5, 11). EPDCs migrate into the subepicardium and contribute to the formation of cardiac fibroblasts and smooth muscle cells (11–14). The potential differentiation of EPDCs into endothelial cells and cardiomyocytes is still topic of debate (15). However, besides their cellular contribution, EPDCs play an important role in enhancing proliferation, maturation, and alignment of cardiomyocytes and as such contribute to the synchronized beating of the heart (16–18). Animal studies in which human adult EPDCs (hEPDCs) were injected after experimentally induced MI showed attenuation of cardiac function which could not be explained by renewal of murine cardiomyocytes, nor by the differentiation of hEPDCs into cardiomyocytes. Other studies showed that transplantation of hEPDCs in the murine heart resulted in increased vascular density and limited post-MI adverse remodeling (6), suggesting that the contribution of hEPDCs was mainly the result of a paracrine effect on the host tissue (6, 19). If hEPDCs improve cardiac function by reducing scar formation via secretion of matrix proteins and participation in structural remodeling of the ECM after MI and which mechanisms are involved is still unclear.

Therefore, the objective of the present study was to investigate the matrix remodeling behavior of hEPDCs both *in vivo* and *in vitro*. The matrix remodeling process involves changes in the ECM driven by both protein synthesis as well as specific enzymes that are responsible for ECM degradation, such as metalloproteinases. Since physical degradation is difficult to determine, we in this study define remodeling as the ability of cells to synthesize ECM protein in the presence of MMPs and TIMPS. It is likely that presence of these enzymes will contribute to degradation. In the *in vivo* approach, we aimed to assess the contribution of hEPDCs to scar remodeling in a non-paracrine fashion. Here, we investigated the presence of human collagen type I, the most abundant fibrillar collagen of the heart, in NOD-SCID mouse hearts after experimentally induced MI and hEPDC injection. To interrogate the mechanisms involved in matrix remodeling, the *in vivo* environment is too complex. Therefore, an *in vitro* modeling approach was chosen. Since, the heart is a continuously beating organ and thereby exposes cyclic strain to its resident cells, the effect of cyclic stain on the matrix remodeling behavior capacity of hEPDCs was investigated. Next, we investigated the contribution of transforming growth factor beta (TGFβ), as one of the key growth factors involved in cardiac repair and fibrosis (20, 21), to the matrix remodeling behavior of hEPDCs.

Our *in vivo* data demonstrate that hEPDCs produce and deposit human collagen in the injured mouse myocardium within the first days after transplantation and are thus able to start the creation of their own matrix environment. Our *in vitro* data further reveal that the expression of collagen type I in hEPDCs is significantly induced by cyclic straining—such as occurring in the native beating heart—and regulated via TGFβ signaling, thereby providing the first insight in hEPDC activation during early stages of matrix remodeling.

## Materials and Methods

### Animal Studies

The *in vivo* contribution of hEPDCs to cardiac ECM remodeling after MI was assessed in a historical dataset of the NOD-SCID murine model of MI and cell transplantation (6, 19). For details on the creation of the MI and the cell transplantation, details can be found in the original manuscripts and the online data supplements (6, 19). In short, to create the MI a permanent ligation using 7.0 suture (Prolene, Johnson and Johnson, New Brunswick, NJ, USA) was created in the left anterior descending coronary artery (LAD). For short term (up till 2 weeks) follow-up, the ligation was located 1 mm caudally from the tip of the left auricle. For long term (6 weeks) experiments, only the frontal branch of the LAD was ligated, since extended survival until 6 weeks is impossible with total LAD ligation. In total 4 × 10^5^ spindle-shaped hEPDCs (passage (P) 2–4) in 20 μl M199 medium were transplanted over 5 injections using a beveled meddle into the ischemic myocardium of the left ventricle and borderzone. To trace the injected hEPDCs, cells were transduced with enhanced green fluorescent protein (eGFP) gene 72 h before transplantation. For the short term experiments cells are transduced with adenoviral vector (hAd/F50.CMV.eGFP, 50 infectious units (IU) per cell. Since adenoviral expression is lost within a few weeks, for long term follow-up hEPDCs were transduced with the lentiviral vector Lv.hPgk.eGFP (12.5 HeLa transducing units/ml per cell) instead of the adenoviral vector.

### Cell Culture

Cultures of hEPDCs were prepared from anonymous human adult right atrial appendages excised during cardiac surgery, as previously described (22, 23). The layer of epicardium was stripped from the auricle, after which the tissue was placed in a gelatin coated culture disk and capped with a round coverslip to prevent the tissue from floating. Cells were cultured in a 1:1 mixture of Dulbecco's modified Eagle's medium (DMEM) (Invitrogen) and Medium 199 (M199) (Invitrogen) 0.5% penicillin (Invitrogen), 0.5% streptomycin (Invitrogen), and 10% heat inactivated fetal calf serum (Invitrogen). Seven days after culturing, when outgrowths of epithelium-like cells were visible the coverslips and remaining tissue were removed. When outgrowth was confluent, the cells were passaged (1:3) to induce spontaneous EMT to create spindle-shaped EPDCs and medium was refreshed every 3 days. Phenotypical profiling of cultured hEPDCs was performed previously to support their epicardial origin (22–24). Biophysical and biochemical stimulation experiments were performed with spindle-shaped hEPDCs from P 4–8 of five different patients. Purity of the hEPDC culture was determined with staining for Wilms tumor-1 (WT1) (Table 1).

**Table 1:** Antibodies used for Immunofluorescent stainings.

**Antigen**	**Source**	**Cat.no**	**Clone**	**Isotype**	**Label**	**Species**	**Dilution**
**Antibodies used for immunofluorescent staining of** ***in vivo*** **material**							
GFP	AB	ab13970		IgG		Chicken	1/1,000
Collagen I	AB	ab138492	–	IgG	–	Rabbit	1/200
Collagen I	SA	C-2456	COL-1	IgG1	–	Mouse	1/100
**Antibodies used for immunofluorescent staining of** ***in vitro*** **material**							
WT1	CA	CA1026	–	IgG	–	Rabbit	1/50
αSMA	SA	a2547	1A4	IgG2a	–	Mouse	1/500
Vinculin	SA	V9264	hVIN-1	IgG1	–	Mouse	1/400
pFAK (Y397)	BD	bd-611723	14/FAKY397	IgG1	–	Mouse	1/1,000
Phalloidin	SA	49409	–	–	Atto 488	Rabbit	1/200
Collagen I	SA	C2456	COL-1	IgG1	–	Mouse	1/100
Collagen III	SA	ab7778	–	IgG	–	Rabbit	1/200
Elastin	AB	ab23747	–	IgG	–	Rabbit	1/100
Fibronectin	SA	F3648	–	IgG	–	Rabbit	1/200
β1-Integrin	SC	sc-53711	TS2/16	IgG1	–	Mouse	1/50
CD44	AB	ab24504		IgG	–	Rabbit	1/100
Mouse IgG1	IG	A21121		IgG1	Alexa Fluor 488	Goat	1/300
Rabbit IgG	IG	A31572		IgG (H+L)	Alexa Fluor 555	Donkey	1/300
Rabbit IgG	MP	A11008		IgG (H+L)	Alexa Fluor 555	Goat	1/200
Mouse IgG1	MP	A21127		IgG1	Alexa Fluor 555	Donkey	1/300
Mouse IgG1	MP	A21240		IgG1	Alexa Fluor 647	Donkey	1/250

*AB, Abcam; SA, Sigma Aldrich; CA, Calbiochem, BD, BD Biosciences; SC, Santa Cruz; IG, Invitrogen; MP, Millipore*.

### *In vitro* Experimental Conditions

For the *in vitro* experiments, cells were plated at a density of 10.000 cells/cm^2^. Cells were allowed to attach for 24 h prior to the onset of the experiments. To elucidate the role of mechanical cues on the remodeling behavior of hEPDCs, cells were cultured under static conditions or exposed to cyclic strain (8%−1 Hz) with the use of the Flexcell system as described below. For biochemical stimulation the culture medium was supplemented with TGFβ3 (0.5 ng/ml) or small molecular inhibitor of the ALK5 pathway, SB-431542 (iALK5, 10 μM) (23). After 48 h of biophysical and/or biochemical stimulators, cells were conducted for further analysis as described below.

### Cyclic Straining of hEPDCs

Cells were seeded on UniFlex® Culture Plates, coated with collagen I (Dunnlab). After 24 h, the cells were subjected to uniaxial cyclic strain (8%) for 48 h by using a Flexcell FX-4,000 Tension straining device (Flexcell, Dunnlab) and compared to static culture (0%). A strain magnitude of 8% and a frequency of 1 Hz were chosen as a suitable strain condition based on the physiological mechanical loads on cells in the myocardial wall (25). Strain experiments were performed with cells derived from five individual human atrial appendages in two independent experiments per subject.

### Immunofluorescent Staining

#### *In vivo* Material

Paraffin sections were immunofluorescently labeled as described previously (26). Briefly, slides were deparaffinized, rehydrated and subjected to heat-induced epitope retrieval with Vector® Antigen Unmasking Solution (Vector). Sections were incubated overnight at 4°C with primary antibodies directed against GFP (Abcam), human collagen I (Abcam), and collagen I (Sigma Aldrich) and were visualized with Alexa-conjugated fluorescent secondary antibodies (Invitrogen) (Table 1). Sections were mounted with ProLong® Gold antifade reagent (Invitrogen) containing 4′,6-diamidino-2-phenylinodol (DAPI).

#### *In vitro* Material

EPDCs cultured on UniFlex® Culture Plates were fixated in 4% paraformaldehyde (Merck) in PBS at RT for 15 min, permeabilized with 0.5% Triton X-100 (Merck) in PBS for 10 min and blocked with 5% BSA in PBS for 30 min. Subsequently, incubation with primary antibodies was performed overnight at 4°C in NET-gel (50 mM Tris, pH 7.4, 150 mM NaCl, 5 mM EDTA, 0.05% NP40, 0.25% gelatin) with 0.5% BSA. The antibodies and their suitable dilutions are listed in Table 1. Incubation for 2 h with Alexa-conjugated fluorescent secondary antibodies (Invitrogen) were used to visualize the primary antibody binding. Nuclear counterstain was achieved by DAPI (1:500) in NET-gel incubation for 5 min. Finally, cover glasses were mounted on microscopic slides with Mowiol.

### mRNA Isolation and Quantitative PCR Analysis

Total RNA was isolated using TriPure and treated with DNAse-I (Qiagen) according to the manufacturer's protocol. Subsequently 250 ng RNA per sample was reverse transcribed into cDNA using the MMLV based cDNA synthesis kit (Bio-Rad). Primers for quantitative PCR (qPCR) were designed with Beacon Designer 7.0 (Premier Biosoft International). Primer sequences and annealing temperatures are presented in Table 2. cDNA samples were subjected to qPCR using IQ™5 SYBR@ Green Supermix (Bio-Rad) and the Bio-Rad IQ™5 detection system (Version 2.0). The average acquired expression cycle threshold (CT) for matrix proteins and modulators and differentiation markers of hEPDCs in different experimental conditions are presented in Table 3.

**Table 2:** Primer sequences.

**Primer**		**Sequence**	**Temp**
**ECM Genes**			
Collagen I	fw	AAT CAC CTG CGT ACA GAA CGG	60
	rv	TCG TCA CAG ATC ACG TCA TCG	
Collagen III	fw	ATC TTG GTC AGT CCT ATG C	60
	rv	TGG AAT TTC TGG GTT GGG	
Elastin	fw	CTG GAA TTG GAG GCA TCG	60
	rv	TCC TGG GAC ACC AAC TAC	
Fibronectin	fw	AAG ACC AGC AGA GGC ATA AGG	60
	rv	CAC TCA TCT CCA ACG GCA TAA TG	
**Matrix Remodelers**			
MMP-1	fw	CGC ACA AAT CCC TTC TAC CC	55
	rv	CTG TCG GCA AAT TCG TAA GC	
MMP-2	fw	ATG ACA GCT GCA CCA CTG AG	60
	rv	ATT TGT TGC CCA GGA AAG TG	
MMP-9	fw	TGG GGG GCA ACT CGG C	60
	rv	GGA ATG ATC TAA GCC CAG	
TIMP-1	fw	TGA CAT CCG GTT CGT CTA CA	49
	rv	TGC AGT TTT CCA GCA ATG AG	
TIMP-2	fw	GGA GGA ATC GGT GAG GTC	60
	rv	AAC AGG CAA GAA CAA TGG	
TIMP-4	fw	GAA TCA TCA CTA CCA TCT	55
	rv	TGC TTC ATA CAG ACA TAA	
**TGFβ** **Pathway**			
Pai	fw	TCT TTG GTG AAG GGT CTG CT	60
	rv	GTG GGT TTC TCC TCC TGT TG	
**Differentiation Markers**			
WT1-isoA	fw	TAT TCT GTA TTG GGC TCC GC	60
	rv	CAG CTT GAA TGC ATG ACC TG	
αSMA	fw	CGT GTT GCC CCT GAA GAG CAT	60
	rv	ACC GCC TGG ATA GCC ACA TAC A	

**Table 3:** Expression cycle threshold Ct) for matrix proteins and modulators and differentiation markers of hEPDCs in different experimental conditions.

**Gene**	**Experimental conditions**				
	**Unstrained**	**Unstrained + TGFβ**	**Strained**	**Strained + iALK5**	**Strained + TGFβ**
*Collagen I*	20.06 ± 1.02	19.35 ± 1.32	19.28 ± 0.77	21.47 ± 0.68	20.25 ± 1.16
*Collagen III*	22.91 ± 1.15	23.14 ± 1.20	22.77 ± 0.78	23.42 ± 1.51	24.25 ± 1.25
*Fibronectin*	22.00 ± 0.68	21.85 ± 0.93	21.90 ± 0.68	23.08 ± 0.90	22.98 ± 1.41
*Elastin*	36.97 ± 2.98	37.84 ± 2.49	38.66 ± 2.25	38.48 ± 2.27	35.90 ± 1.35
*MMP-1*	22.74 ± 1.87	23.96 ± 2.00	23.82 ± 1.45	23.39 ± 1.71	22.43 ± 1.33
*MMP-2*	23.00 ± 0.93	22.80 ± 1.95	22.89 ± 1.83	23.93 ± 1.16	23.69 ± 1.87
*MMP-9*	34.20 ± 3.74	35.41 ± 1.71	35.42 ± 1.60	35.22 ± 1.78	34.53 ± 1.11
*TIMP-1*	21.65 ± 0.94	21.87 ± 0.83	21.67 ± 0.74	21.84 ± 0.92	–
*TIMP-2*	23.31 ± 0.38	23.60 ± 0.84	23.28 ± 0.55	23.99 ± 0.75	23.66 ± 0.35
*TIMP-4*	33.03 ± 0.69	31.94 ± 0.56	32.72 ± 0.68	33.71 ± 0.72	–
*Pai-1*	22.73 ± 1.30	22.08 ± 1.30	21.76 ± 1.01	23.75 ± 1.30	22.92 ± 1.81
*WT1-isoA*	29.28 ± 2.35	30.38 ± 2.58	30.43 ± 2.46	29.63 ± 3.43	32.07 ± 2.42
α*SMA*	24.93 ± 1.16	24.24 ± 1.70	24.46 ± 1.25	26.71 ± 0.59	25.58 ± 1.56

*The cycle threshold Ct) for each gene varied over the different experimental conditions and between patients. Not applicable Ct values were set at a value of 40. The table represents mean Ct values + SD. Genes that are highly expressed gave a Ct value <20, moderate expression was shown by Ct values of 20–26. Low and hardly expressed genes gave a high Ct value of 26–34 and 34–40, respectively*.

### Gelatin Zymography

Gelatin zymography was performed to semi-quantitatively determine the proteolytic activity of metalloproteinases (MMP)-2 and−9 in media samples, as described previously (27, 28). Total protein concentrations were determined by BCA Protein Assay (Pierce), before 5 mg protein was separated on a 10% sodium dodecyl sulfate-polyacrylamide gel (SDS-page). Gels were rinsed in 2.5% Triton X-100 to remove the SDS and incubated overnight in zymography substrate buffer (50 mM Tris/HCL, 0.05% (w/v), calcium chloride (CaCl_2_); pH8.5) to activate the MMPs. Gelatinolytic activity was visualized with Coomassie Blue solution, followed by destaining in destainer solution. The amount of active or latent MMP-2 (resp. 66 and 72 kDa) and active or latent MMP-9 (resp. 86 and 92 kDa) was visualized using Proxima C16 Phi+ (Isogen). Intensity of the bands (INT^*^mm^2^ with global background correction) was determined using Quantity One image analysis software (*n* = 4).

### Statistical Analysis

All data is presented as mean ± SD. Quantification of qPCR data was performed with comparative threshold cycle (Ct) method (ΔΔCt) of Livak et al. (29), using GAPDH as internal control gene and the average of the unstrained samples as calibrator. Data is presented as fold change in scatter-plots and each dot on the scatter-plot represents the observation of cells derived from one patient (total *n* = 5 patients). Statistical analysis of the results was accomplished by the non-parametric Mann-Whitney test to compare the grouped samples. For statistical analysis for multiple comparisons a one-way ANOVA with Bonferoni correction for multiple comparisons was used when equal variances were assumed or a Kruskall-Wallis assuming non-equal variances, with Dunn's *post-hoc* test. Zymographic results were quantified by comparing Density (INT) of the MMP bands. Statistical analysis of zymography data was performed using a Kruskall-Wallis test, assuming non-equal variances, with Dunn's *post-hoc* test for multiple comparisons. Statistical significance was considered when *P* < 0.05. Graphics and statistical analysis were performed using the Graphpad Prism 6 software package (Graphpad Software).

## Results

### *In vivo* Evaluation of the Production of Collagen I by hEPDCs After MI

**Figure 1:**
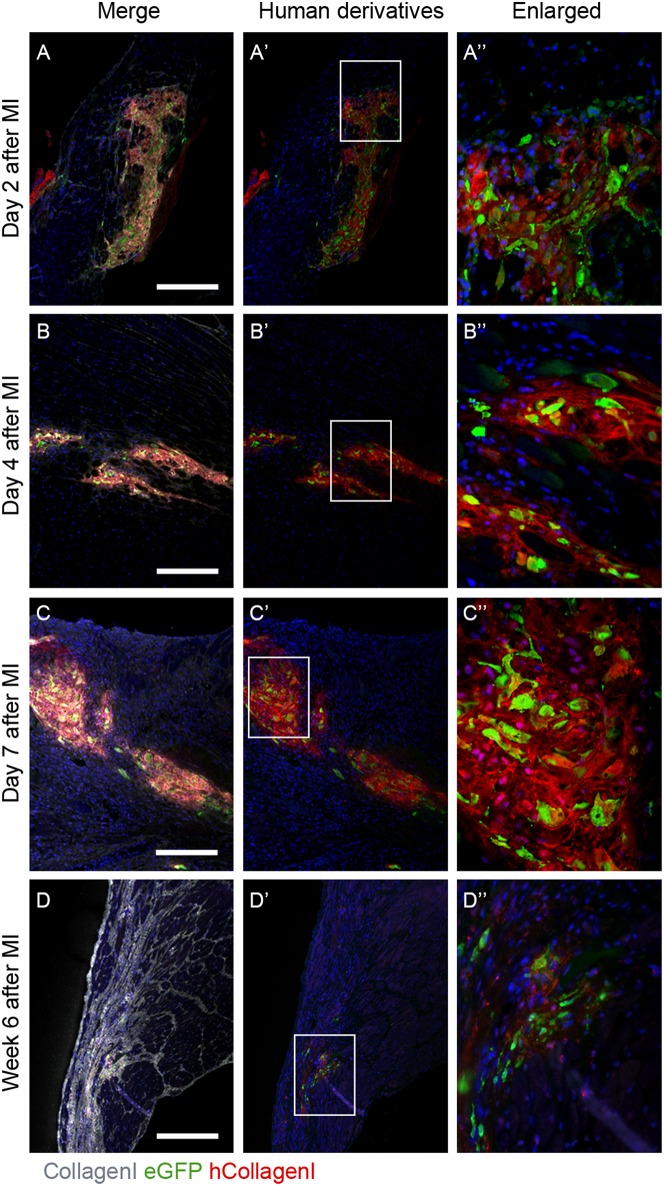
Human collagen type I is present in the infarcted NOD-SCID mouse heart after hEPDC injection. **(A)** Two days after injection of hEPDCs (green) in the mouse infarcted heart, human collagen I (red) is present. The expression of human collagen I (red) increased at day 4 **(B)** and 7 **(C)** after MI. **(D)**. After 6 weeks, hEPDCs (green) were still present in the infarcted mouse heart. At this time point, the total amount of collagen I (white) was prominent, while the amount of human collagen type I (red) was markedly reduced. Images **(A'–D')** represent location of hEPDCs (green) and human collagen I (red) and images **(A”–D”)** represent enlargements of images **(A'–D')**. Scale bar 250 μm.

It was previously reported that in NOD-SCID mice after experimentally induced MI and transplanted with GFP-labeled hEPDCs show preservation of the left ventricular function accompanied with increased wall thickness and vascular density (6). To investigate the ability of transplanted hEPDCs to contribute to matrix synthesis in a non-paracrine fashion, we determined the presence of human collagen type I, the predominant cardiac ECM component, by immunofluorescent staining at several time points in the first week after injection and at 6 weeks post-MI. Already 2 days after cell injection, the presence of human collagen I was observed in the NOD-SCID murine heart (Figures 1A–A”). The collagen type I protein was found both intra- and extracellular, indicating both production and deposition of human collagen within the mouse heart by injected hEPDCs. During the first 7 days post MI, the protein was stably expressed (Figures 1B–B”,C–C”).

Six weeks after injection, hEPDCs still resided within the injured mouse myocardium, as shown by the presence of eGFP positive cells (Figures 1D',D”). The total amount of collagen type I present in the myocardium was more intense and prominent compared to earlier time points, while the expression of human collagen type I was strongly reduced (Figure 1D). These *in vivo* data suggest that hEPDCs have an early response to the infarcted mouse heart by creating their own collagen type I niche.

### Cyclic Strain Induces Collagen I Expression by hEPDCs

The first signs of collagen I synthesis and thereby contribution to matrix deposition by hEPDCs, were already visible 48 h after transplantation into the NOD-SCID mouse heart. Therefore, this early time point in matrix remodeling was chosen for the duration of application of cyclic strain *in vitro*. To secure that hEPDCs are sensitive for mechanical cues, the presence of the F-actin and the mechanosome proteins pFAK and vinculin were investigated (Figure S1).

**Figure 2:**
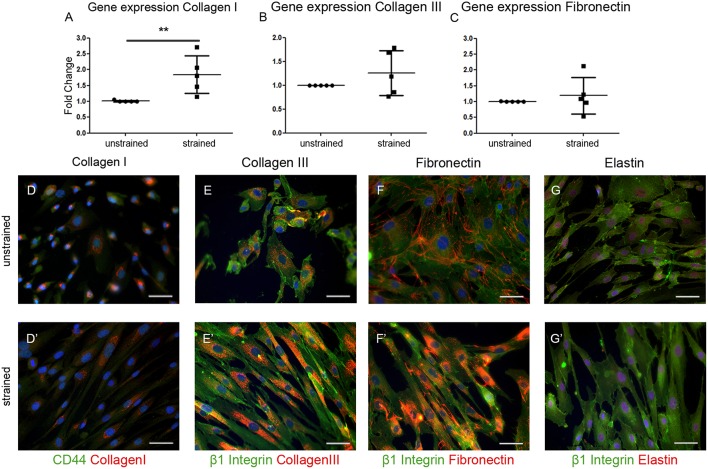
Extracellular matrix (ECM) proteins are synthesized by hEPDCs. Validation of qPCR data on the expression of ECM genes collagen I **(A)** collagen III **(B)**, and fibronectin **(C)** revealed induction of these genes under exposure to uniaxial cyclic strain. These data are represented as mean fold increase ± SD. Since the average ΔCt of the unstrained samples was used as calibrator, the fold change (2^Δ*ΔCt*^) of the unstrained group is close to 1.0. Representative fluorescent images confirm the biosynthesis of these ECM proteins collagen I **(D)**, collagen III **(E)**, fibronectin **(F)**, and elastin **(G)** in red, co-stained by cell surface markers CD44 **(D′)**, and β1 Integrin **(E′-G′)** both in green. **P < 0.01. Scale bar 50 μm.

To gain more insight into the contribution of hEPDCs to matrix production, next to collagen type I, other cardiac matrix components were investigated. Analysis of ECM proteins at the mRNA level showed that under static conditions, hEPDCs express collagen I, collagen III, and fibronectin with Ct values of approximately 20, 23, and 22 (Figures 2A–C; Table 3), respectively, while expression of elastin was nearly absent (Ct value of 37).

Double immunofluorescent staining with either the cell-surface marker CD44 or β1 integrin to visualize the cell boundaries confirmed presence of collagen I, collagen III, and fibronectin inside the unstrained cells (Figures 2D–F). Although elastin was hardly expressed on mRNA level, staining was present in close proximity to the nuclei in the unstrained samples (Figure 2G). Exposure of the cells to cyclic strain for 48 h increased the expression of collagen I mRNA significantly (*p* = 0.0079), while application of cyclic strain only showed a trend toward up-regulation for the other ECM genes. On protein level this strain induced gene expression resulted in re-localization of collagen I and -III protein from near the nucleus to more divided throughout the cell (Figures 2D',E'). Fibronectin expression was clear at the border of the cell in unstrained samples, but after exposure to cyclic strain the protein was distributed throughout the cytoplasm (Figure 2F'). The expression pattern of elastin was not changed by application of strain (Figure 2G'). Altogether, these data show that hEPDCs are able to synthesize ECM proteins and that exposure to biomechanical cues *in vitro* induces relocalization of the protein, which might be indicative for new synthesis.

### ECM Remodeling Capacity of hEPDCs Is Not Affected by Cyclic Strain

In later stages after MI, the ability of cells to remodel the ECM is of great importance for cardiac repair and the maintenance of cardiac function. Since matrix metalloproteinases (MMPs) play an important role in matrix degradation and remodeling we analyzed their presence. Interestingly, MMP-1 and−2 were expressed by unstrained hEPDCs (Figure S2; Table 3), while MMP-9 was hardly present (mean Ct-value around 34 in unstrained hEPDCs). Exposure to cyclic strain for 48 h revealed no clear changes in expression of MMPs probably due to large variation between patients (Figure S2; Table 3).

Since not only the expression of MMPs, but also their enzymatic activity plays a role in matrix remodeling, we determined MMP-2 and−9 activity in culture media. Zymography on plain media samples showed already expression of latent MMP-2 and−9 and active MMP-2 (Figure S2D), which is caused by the presence of serum in the culture media which contains MMPs. Analysis of the zymography data showed that the levels of both inactive and active MMP-9 and MMP-2 were increased in hEPDC-derived media samples compared to culture media alone (Figure S2D). Semi-quantitative analysis was performed and corrected per experiment for the INT of the unstrained samples, due to high variance between experiments probably caused by the amount of MMPs already present in the plain media. Analysis revealed stable levels of latent and active MMP-9 between unstrained and strained media samples (Figures S2E,F). In all hEPDC conditioned media samples all three forms of MMP-2, latent, active intermediate MMP-2 form (64 kDa) and active MMP-2 (66 kDa) were present. In the time span of 48 h application of cyclic strain did not induce any change in activity of MMP2 (Figure S2).

MMP activation is regulated by the tissue inhibitors of metalloproteinases (TIMPs). TIMP-1 and−2 were moderately expressed by hEPDCs, while the expression of TIMP-4 was low (mean Ct-value of 33.0 in unstrained hEPDCs (Figure S2; Table 3). The application of strain for 48 h did not influence the expression of any of the TIMPs (Figure S2).

### Cyclic Strain Induces the TGFβ Signaling Pathway

Cytokines and growth factors are excessively produced by the injured heart and mechanical cues can modify and activate cytokines and growth factors from their reservoirs within the ECM (30). Although, several signaling pathways are involved in tissue repair, pathogenesis and matrix remodeling, TGFβ is the key factor in matrix production for tissue repair and is rapidly induced and activated in the injured heart (24, 31–33). Furthermore, the TGFβ-pathway plays an important role in the activation and the differentiation of hEPDCs (23, 24). Therefore, the role of TGFβ as a biochemical cue on the contribution of hEPDCs on matrix production and remodeling was investigated.

**Figure 3:**
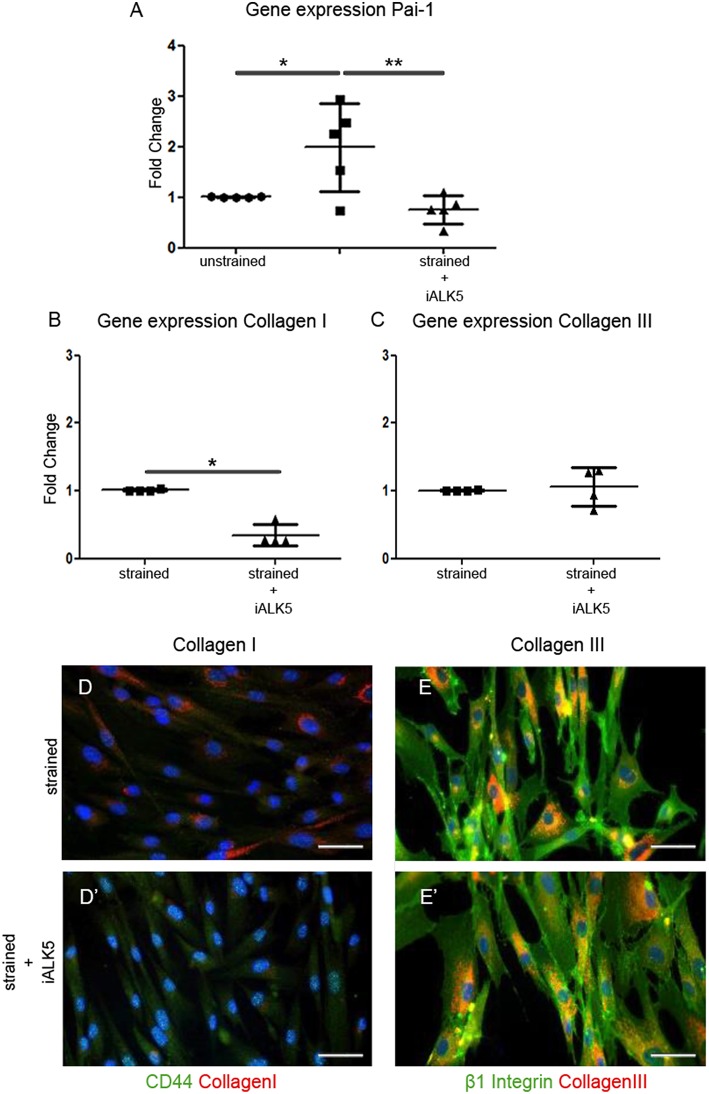
TGFβ signaling is induced in hEPDCs exposed to uniaxial cyclic strain. Gene expression of unstrained and strained hEPDCs showed that the expression of *Pai-1* was increased by application of strain **(A)**. Gene expression of hEPDCs exposed to strain in the presence of iALK5 showed a significant decrease in *Pai-1*. Inhibition of TGFβ signaling also resulted in a significant downregulation of collagen I **(B,D,D')**, while no significant change was found in the expression of collagen III **(C,E,E')**. The data are represented as mean fold change ± SD. *P < 0.05, **P < 0.01.

To determine whether the TGFβ signaling pathway is involved in the strain induced collagen I production, the effect of cyclic strain on one of the major downstream TGFβ targets and important modulator of matrix/tissue remodeling, gene platelet activator inhibitor-1 (Pai-1) was investigated. The expression of Pai-1 increased almost 2-fold after application of cyclic strain (*P* < 0.05) and reduced 1.3-fold compared to the unstrained sample when TGFβ signaling was blocked by addition of the ALK5 kinase inhibitor (SB-431542) to the media of the strained samples (*P* < 0.01) (Figure 3A). To gain more insight in the involvement of the TGFβ signaling pathway in strain induced collagen production, the effect of cyclic strain in combination with SB-431542 on mRNA level was investigated. Analysis showed that under strained conditions combined with blocking of the TGFβ signaling pathway, the expression of collagen I decreased with 65% (*P* < 0.05) (Figure 3B), while the expression of collagen III was unaffected (Figure 3C). Double immunofluorescent staining with either the cell-surface marker CD44 or β1 integrin to visualize the cell boundaries confirmed this change of collagen I and the stability of collagen III inside the strained cells treated with ALK5 kinase inhibitor (Figures 3D,D',E,E'). These data suggest that strain induces signaling via the TGFβ/ALK5 pathway.

In addition, the influence of TGFβ signaling on presence of MMPs and TIMPs was analyzed. Zymography revealed no significant effect of TGFβ addition on the activity of MMP2 or MMP9 nor does the inhibition of the TGFβ/ALK5 signaling pathway (Figure S3). On gene level the same trend for MMP mRNA expression was shown in unstrained samples supplemented with TGFβ and strained samples (Figure S3).

**Figure 4:**
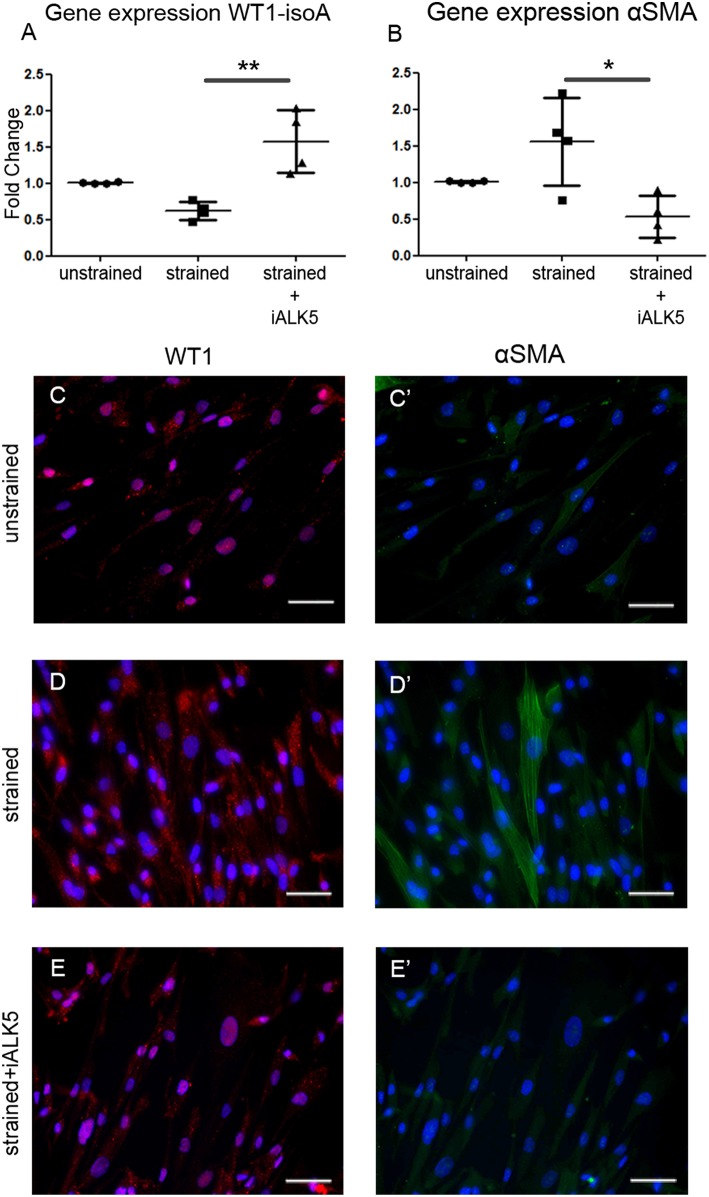
Application of cyclic strain induces changes in differentiation markers in hEPDCs and ALK5 is required for this effect. Quantification of *WT1* isoform A mRNA expression decreased after application to strain and increased again by simultaneous exposure to strain and iALK5 **(A)**. The onset of differentiation was also shown by the increase in αSMA mRNA in strained hEPDCs, which was inhibited in the presence of iALK5 **(B)**. **(C–E)** The process of differentiation was confirmed by staining for WT1 and αSMA **(C'–E')** as shown by translocation of the WT1 expression from the nuclei to the cytoplasm and the presence of αSMA after exposure to cyclic strain. The data is represented as represented as mean fold change ± SD. *P < 0.05, **P < 0.01. Scale bar 50 μm.

TGFβ signaling is known for its role in the differentiation of hEPDCs toward fibroblasts and/or smooth muscle cells. Fibroblasts have a role in the matrix biosynthesis as major collagen producers (34, 35), therefore the differentiation status of hEPDCs was evaluated. Although the epicardial marker WT1 was present both at mRNA and protein level in unstrained as well as strained hEPDCs, application of uniaxial cyclic strain reduced the expression of WT1 by 38% (n.s.) (Figure 4A). Most of the hEPDCs showed nuclear expression of WT1 protein (Figure 4C), while under strain conditions WT1 was also present in the cytoplasm (Figure 4D). The expression of this epicardial marker increased significantly when strain was applied in the presence of iALK5 (Figure 4A) and was accompanied with translocation of WT1 from the cytoplasm to the nucleus (Figure 4E). When differentiating into fibroblast and/or smooth muscle cell, hEPDCs start to form actin filaments which is accompanied by an increase in alpha smooth muscle actin (αSMA) mRNA (Figure 4D). Although qPCR revealed a slight increase in αSMA expression under strain conditions (n.s.), on protein levels hEPDCs display more clear actin fibers compared to unstrained cells (Figures 4C',D'). The expression of αSMA, was significantly downregulated in cells exposed to both strain and iALK5 (Figure 4B). This down regulation was confirmed by fluorescent staining, revealing absence of actin fibers (Figure 4E'). These data suggest that the ALK5 dependent TGFβ signaling induces hEPDC differentiation into a matrix producing mesenchymal cell phenotype.

To elucidate whether mechanical cues, the excessive presence of TGFβ in the injured heart, or the combination of both is the trigger for matrix production and remodeling ability by hEPDCs, all three conditions were compared. Data revealed that addition of an excess of TGFβ to unstrained cells or combining the application of strain simultaneously with addition of TGFβ for 48 h had no significant effect on the expression of matrix proteins (Figure S4) or on the differentiation potential of hEPDCs compared to cells only exposed to strain (Figure S4). This lack of synergistic effect on ECM production with combining cyclic stretching and TGFβ was previously shown in dermal fibroblasts (36).

## Discussion

Cardiac reparative therapies for the diseased heart aim at functional repair of myocardial contractibility to prevent heart failure. The challenge is to not only create new contractile units and vessels, but to also strive for favorable remodeling or even replacement of the fibrotic matrix, thereby fulfilling the basic requirements for long term improvement of heart function. In this study, we investigated the ability of hEPDCs to secrete human collagen I in the myocardial infarction microenvironment as first step in understanding the contribution of hEPDCs to scar composition in a non-paracrine manner. Additionally, an *in vitro* approach was used in which cells were exposed to cyclic strain and exogenous stimulation or inhibition of TGFβ signaling to start unraveling the mechanisms involved in the matrix remodeling capacity of hEPDCs. Our key findings reveal that (1) hEPDCs are able to produce collagen I in the acute phase after MI, (2) cyclic straining induces collagen type I by hEPDCs, (3) TGFβ/ALK5 signaling is an important driver in hEPDC induced matrix synthesis.

### Human Collagen I Is Synthesized in the Injured Mouse Heart

As previous shown, transplantation of hEPDCs into the ischemic myocardium of NOD-SCID mice was able to attenuated ventricular remodeling via a paracrine stimulatory effect (6). Characterization of these transplanted hEPDCs *in vivo* revealed expression of αSMA, von Willebrand factor (vWF), sarcoplasmic or endoplasmic reticulum Ca^2+^-ATPase (SERCA2a) and voltage-gated sodium channel (α-subunit; SCN5a), suggesting a myoendothelial phenotype (6). Our data indicate that hEPDCs not only contribute to ventricular remodeling via secretion of growth factors and cytokines, but are also able to create their own matrix environment when transplanted in the acute phase after MI. The creation of this human microenvironment is only temporary, since human collagen is almost absent in the infarcted area 6 weeks after transplantation suggesting active remodeling of the human matrix during myocardial wound healing. This remodeling could be induced by the hEPDCs themselves or by the high expression levels of remodeling proteins in the injured mouse heart (37). This is, to the best of our knowledge, the first study describing that adult hEPDCs directly contribute to matrix production in the early response phase after MI, although indirect paracrine effects of hEPDCs on matrix remodeling have been speculated (10, 38) and matrix production by EPDCs is described in the embryonic mammalian heart or by endogenous EPDCs in mouse models (8, 39). The production of collagen type I by hEPDCs can positively regulate cardiac function and repair. By producing human collagen I, hEPDCs create their own local supportive environment to maintain cellular structure and function, thereby favoring cell survival which will enhance repair. Furthermore, collagen I provides tensile strength and structural integrity, which creates resistance to stretch and deformation during each contraction. In the injured heart this prevents the myocardial wall from rupture and contributes to preservation of contractility (40). Moreover, the deposition of epicardial-derived ECM could promote wound healing and thereby might direct cardiac regeneration (41). The negative impact of human collagen I production by EPDCs on cardiac regeneration may arise by the fact that collagen type I is the major fibrillar collagen present in the mature scar, and therefore might have negative consequences for repair, remodeling and functional competence of the heart on the long term (3, 38). The production of human collagen type I by the hEPDCs could be a response to the inflammatory cytokines or other remodeling factors present after MI. However, we have shown that hEPDCs are mechanosensitive, and our *in vitro* data suggest that mechanical stress contributes to the increase in collagen type I deposition. Therefore, we think that the *in vivo* response of these cells, especially during the early phase post MI when a definitive scar is not yet formed, is a combination of mechanical stress and inflammatory cytokines. Further research is needed to unravel if collagen production by hEPDCs in the early response phase after MI may trigger the transition from reparative fibrosis to malignant scaring at late remodeling phases.

### EPDCs Respond to Environmental Mechanical Stimuli *in vitro*

Given the complexity of the environment in the injured heart, we used an *in vitro* approach to elucidate how the different environmental stimuli interplay and guide cellular responses. This systematic and reductionist approach *in vitro* was followed to gain understanding in the contribution of cyclic strain and biochemical stimulation. Our data reveal that adult hEPDCs are able to sense and respond to mechanical cues from their environment. Indeed, when exposed to uniaxial cyclic strain, EPDCs reorient perpendicular to the stretch direction, a behavior also observed in several mesenchymal cell types, known as “strain avoidance” (42, 43) (Figure S1). In response to cyclic strain the TGFβ signaling pathway in hEPDCs is induced and cells thereby change matrix synthesis and their differentiation profile. These findings confirm the suggestion of mechanosensitivity of epicardial cells by Andrés-Delgado et al. who reported that during cardiac development the heart beat drives the epicardium formation from the pro-epicardial organ (44).

To date, little is known about matrix modulation by adult mammalian EPDCs and the influence of mechanical stimulation on this process. EPDCs display stromal cell properties and they are progenitors of cardiac fibroblasts (9, 10). Cardiac fibroblasts are key players in matrix remodeling in the developing, adult and diseased heart (34, 35) and application of cyclic mechanical strain increase the synthesis of collagens and fibronectin by cardiac fibroblasts (45–48). In this research we investigated the early matrix remodeling response, after 48 h and therefore it is difficult to draw definitive conclusions regarding the effect of mechanical stimuli on differentiation outcome. After 48 h of exposure to cyclic strain, hEPDCs still express WT1 and they lack expression of elastin, which suggest that these hEPDCs are different from cardiac fibroblasts. A recent study using cardiac fibroblasts, suggested that strain-induced matrix production could precede differentiation toward myofibroblasts, since cardiac fibroblasts are adapted to the physiological cyclic strains or have a memory that reminds them how to coop with mechanical stimuli, which prevents them from myofibroblast activation (46). This hypothesis is also plausible for hEPDCs, suggesting that cyclic strain causes the onset of transformation toward a matrix producing mesenchymal cell (35, 48, 49).

### TGFβ-Signaling Is Part of Strain-Induced Matrix Reorganization Ability of hEPDCs

As also shown in this study, mechanical stimulation has an important impact on the activity of TGFβ (30). Next to mechanical stimulation, proteases like MMP-2 and MMP-9 are able to activate TGFβ (31). Interestingly, TGFβ also suppresses protease activity by inhibiting MMP expression and the induction of synthesis of protease inhibitors like Pai-1 and TIMPs (32, 33) and Pai-1 induction is shown in our study.

We found no additional effect when combining mechanical cyclic stimuli and addition of exogenous TGFβ on matrix synthesis and remodeling by hEPDCs. The ability of cells to respond to experimental conditions is dependent on the activation status of the cells prior to start of the experiment (49). The lack of response of hEPDCs to exogenous TGFβ could be due to already elevated TGFβ levels as inhibiting the pathway did influence the ability of hEPDCs to respond (35, 48). Elevation in TGFβ levels could be induced by cell passaging, which in cardiac fibroblasts leads to differentiation into myofibroblasts after the third passage (50). In addition, presence of TGFβ will influence the activation and differentiation profile of the cells at the start of the experiments, which might also explain the large inter-patient variation we observe. Therefore, it is important to determine the heterogeneity in activation status and differentiation profile prior to start of the experiment. For translation to clinical application it is important to gain more insight in the link between the activation and differentiation profile of hEPDCs and the contribution of these cells to matrix synthesis and remodeling both *in vivo* and *in vitro*.

### Implications for Cardiac Repair Therapy

Given its developmental contribution in cardiomyogenesis (15), the epicardium is a highly interesting cell layer in the context of cardiac repair after ischemic injury. Therefore, EPDCs offer promising outlooks for cell-based cardiac therapy; either via exogenous application, or via endogenous reactivation (6, 9, 10, 15, 18, 19, 38). EPDCs can contribute to cardiac repair by (1) providing new cells via differentiation into cardiac fibroblasts and smooth muscle cells and (2) paracrine signaling. The latter process leads to production of essential proteins and influences myocardial growth, which can further guide cardiac repair after injury and maintain the beating rate of cardiomyocytes (15, 32).

The production of collagen I by hEPDCs directly after MI is crucial for preserving myocardial function, since inhibition of collagen deposition leads to worsening of cardiac dilation and heart dysfunction (37). ECM production by EPDCs during MI healing can both be positive or negative for cardiac repair, and the right balance in the production and remodeling of the cardiac ECM is of great importance. Therefore, it is important to explore how pathological myocardial conditions contribute to physical and paracrine properties of hEPDCs in more detail. This future research will provide new insights, which might lead to new treatment modalities for cardiac repair.

## Conclusion

In summary, we show that hEPDCs are able to synthesize and excrete collagen I after MI and thereby create their own extracellular environment. To elucidate the role of the pathological environment in the hEPDC response to cardiac injury, we proposed an *in vitro* set-up to elucidate the role of mechanical stimuli and/or TGFβ signaling as biochemical cue. We revealed that mechanical stimulation is able to trigger matrix reorganization, mainly through collagen I production, via the TGFβ/ALK5 signaling pathway. The *in vitro* approach enables unraveling the ability of hEPDCs to create and remodel the cardiac matrix in response to injury. These insights have impact on future studies on the contribution of hEPDCs to reverse or adverse remodeling and better understanding of this process might tailor new treatment modalities.

## Ethical Statement

All animal procedures to achieve this dataset were approved by the Animal Ethics Committee of the Leiden University and conformed the Guide for the Care and Use of Laboratory Animals (National Institutes of Health publications No. 85-23, Revised 1996).

## Author Contributions

NB, SD, CB, and MG contributed substantially to the conception and design of the work. While NB, SD, BK, AS, CB, and MG contributed to the acquisition, analysis, and interpretation of data. NB and SD drafted the work and CB and MG revised it critically for intellectual content. All authors approved of the final version to be published and agreed to be accountable for all aspects of the work in ensuring that questions related to the accuracy or integrity of any part of the work are appropriately investigated and resolved.

### Conflict of Interest Statement

The authors declare that the research was conducted in the absence of any commercial or financial relationships that could be construed as a potential conflict of interest.
