# Supplementary material for: In vivo and in vitro Approaches Reveal Novel Insight Into the Ability of Epicardium-Derived Cells to Create Their Own Extracellular Environment
Source: Front Cardiovasc Med. 2019 Jun 19;6:81. doi: 10.3389/fcvm.2019.00081 (PMC6594358; doi:10.3389/fcvm.2019.00081)
Supplement: Supplementary file 1 [file Image_1.pdf]

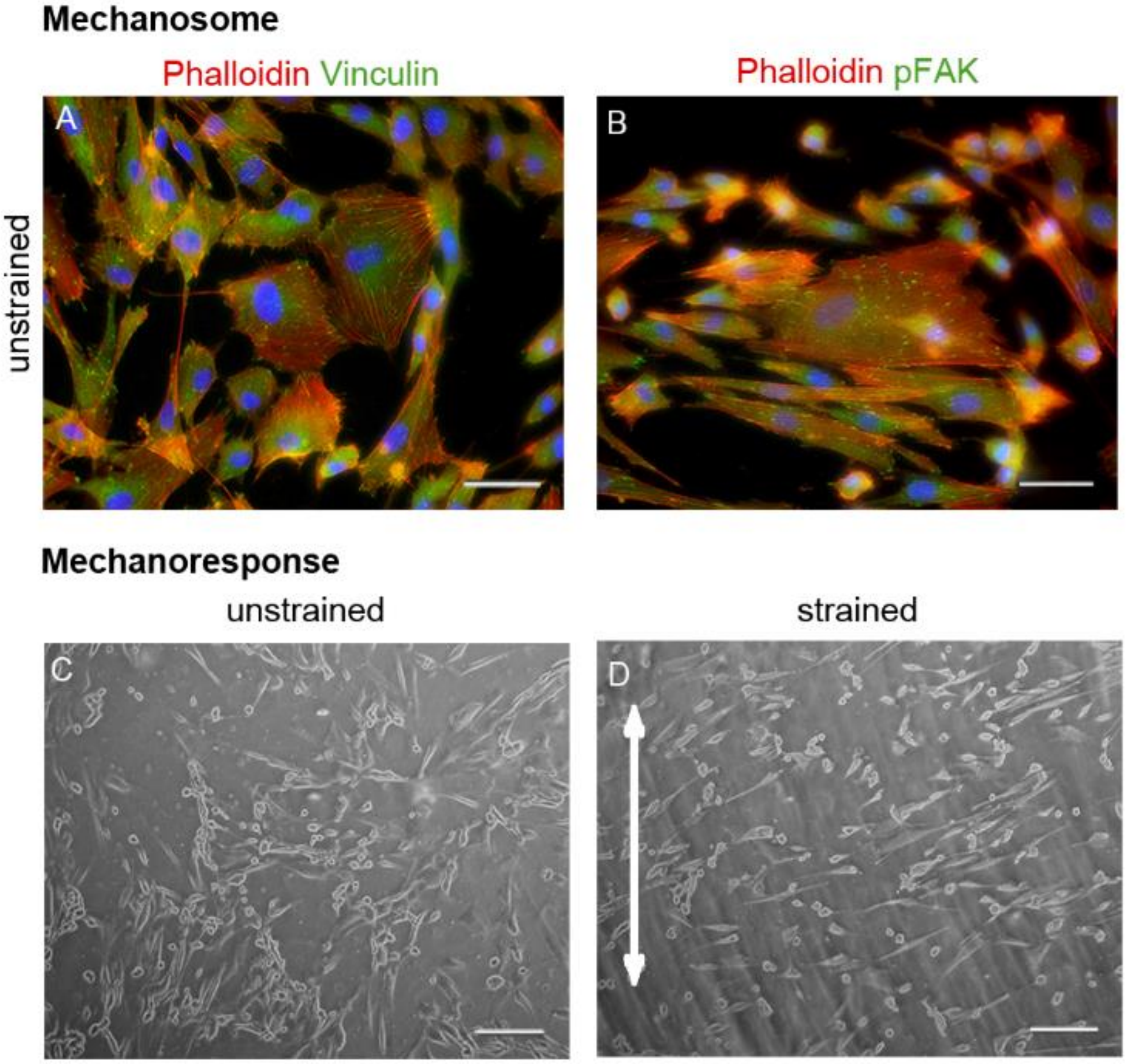

Figure S1. Focal adhesion formation and cytoskeleton development in hEPDCs. Immunofluorescent images of phalloidin (A,B) show the presence of the cytoskeleton in unstrained hEPDCs. Vinculin (A) and phosphorylated focal adhesion kinase (pFAK) (B) show the presence of focal adherent proteins in unstrained hEPDCs, components of the structural complex of the mechanosome. EPDCs revealed strain avoidance response (C,D) when exposed to uniaxial cyclic strain. Scale bar 50µm (A,B) and 500µm (C,D).
