# Supplementary material for: In vivo and in vitro Approaches Reveal Novel Insight Into the Ability of Epicardium-Derived Cells to Create Their Own Extracellular Environment
Source: Front Cardiovasc Med. 2019 Jun 19;6:81. doi: 10.3389/fcvm.2019.00081 (PMC6594358; doi:10.3389/fcvm.2019.00081)
Supplement: Supplementary file 2 [file Image_2.pdf]

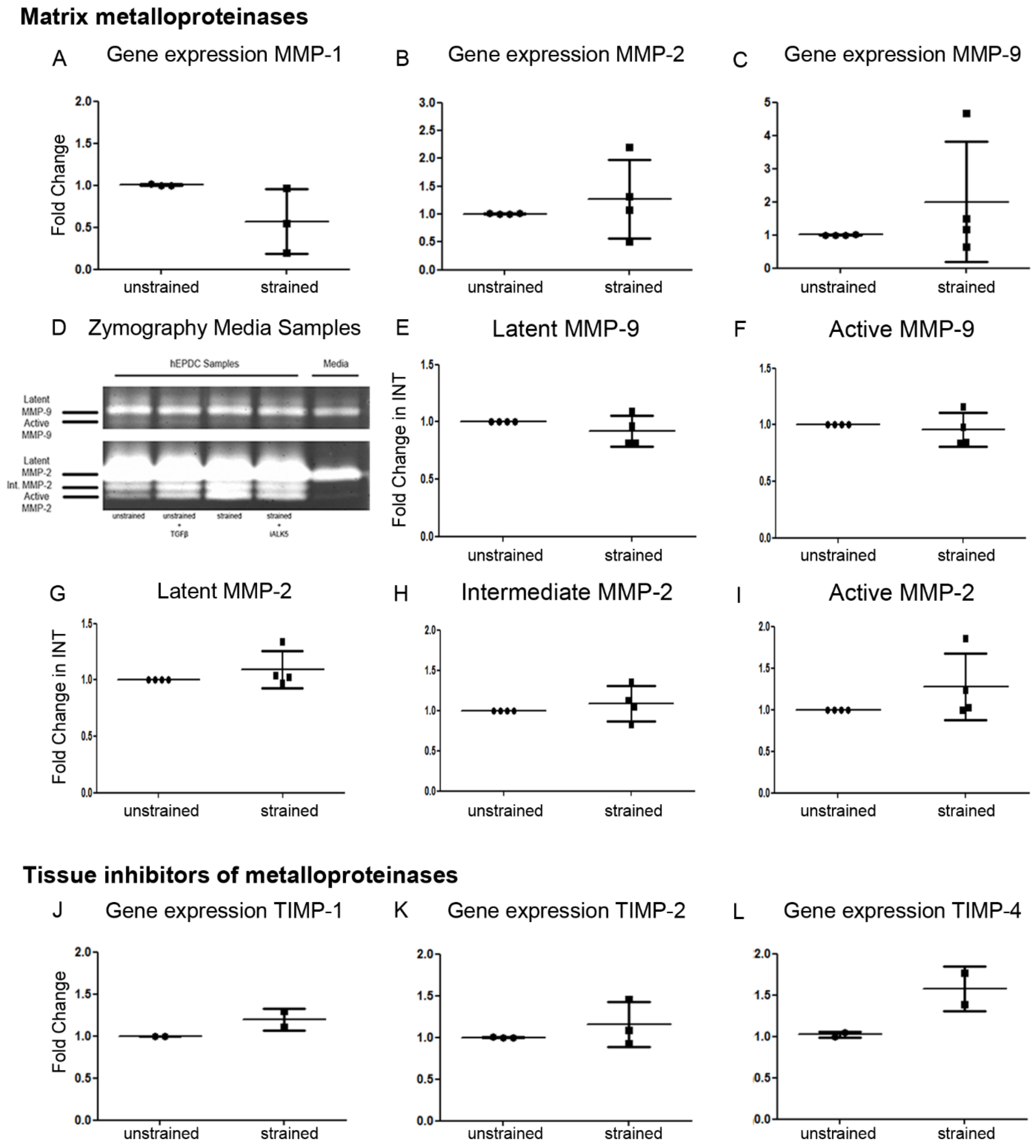

Figure S2. Matrix modulators and their inhibitors are expressed by hEPDCs. The mRNA levels of MMP-1 (**A**), MMP-2 (**B**) and MMP-9 (**C**) were not influenced by exposure of the cells to uniaxial cyclic strain. Zymography results of MMPs in media samples (**D**) and their quantification (**E-I**) confirmed that there were no changes under exposure to cyclic strain. There was no difference in the expression of inhibitors of matrix remodeling factors TIMP-1 (**J**) and TIMP-2 (**K**) under exposure to strain, while TIMP-4 (**L)** was slightly induced although not significantly. Data are presented as mean fold change ± SD. In the analysis of the zymography samples the data were compared per experiment and corrected for the INT of the unstrained samples, represented as fold change in INT as 1.0 resulting in the very low SD-values in the gene expression fold change of the unstrained samples for *TIMP-1*, *TIMP-2* and all *MMPs*.
