# Supplementary material for: In vivo and in vitro Approaches Reveal Novel Insight Into the Ability of Epicardium-Derived Cells to Create Their Own Extracellular Environment
Source: Front Cardiovasc Med. 2019 Jun 19;6:81. doi: 10.3389/fcvm.2019.00081 (PMC6594358; doi:10.3389/fcvm.2019.00081)
Supplement: Supplementary file 3 [file Image_3.pdf]

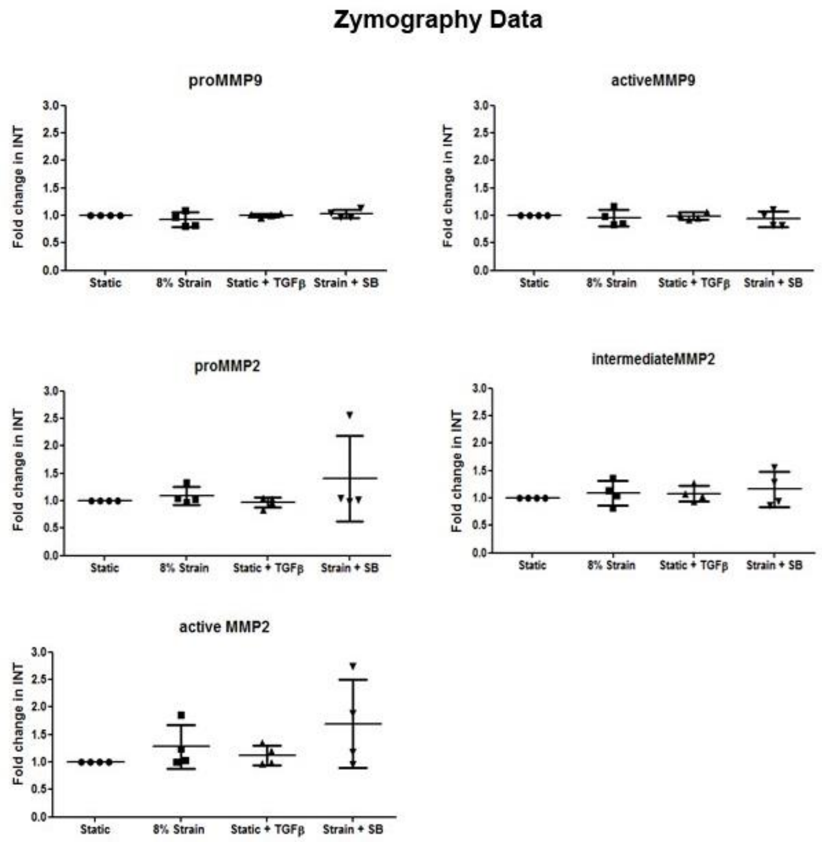

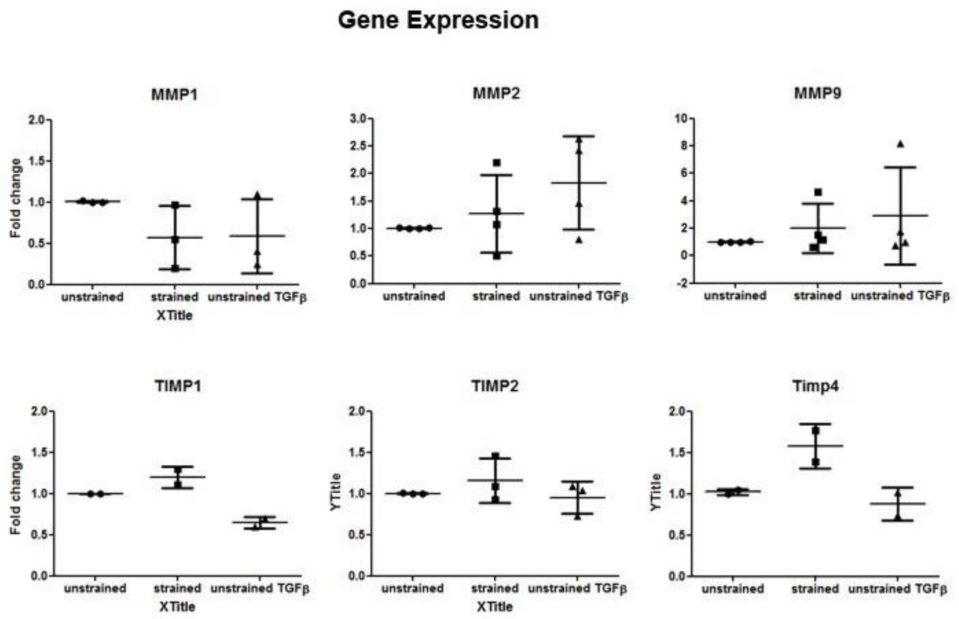

Figure S3. Data is presented as changes compared to unstrained conditions. There were no significant changes in expression of *MMPs* and *TIMPs* under TGFβ (0.5ng/ml) stimulation compared to unstrained conditions.
