# Supplementary material for: In vivo and in vitro Approaches Reveal Novel Insight Into the Ability of Epicardium-Derived Cells to Create Their Own Extracellular Environment
Source: Front Cardiovasc Med. 2019 Jun 19;6:81. doi: 10.3389/fcvm.2019.00081 (PMC6594358; doi:10.3389/fcvm.2019.00081)
Supplement: Supplementary file 4 [file Image_4.pdf]

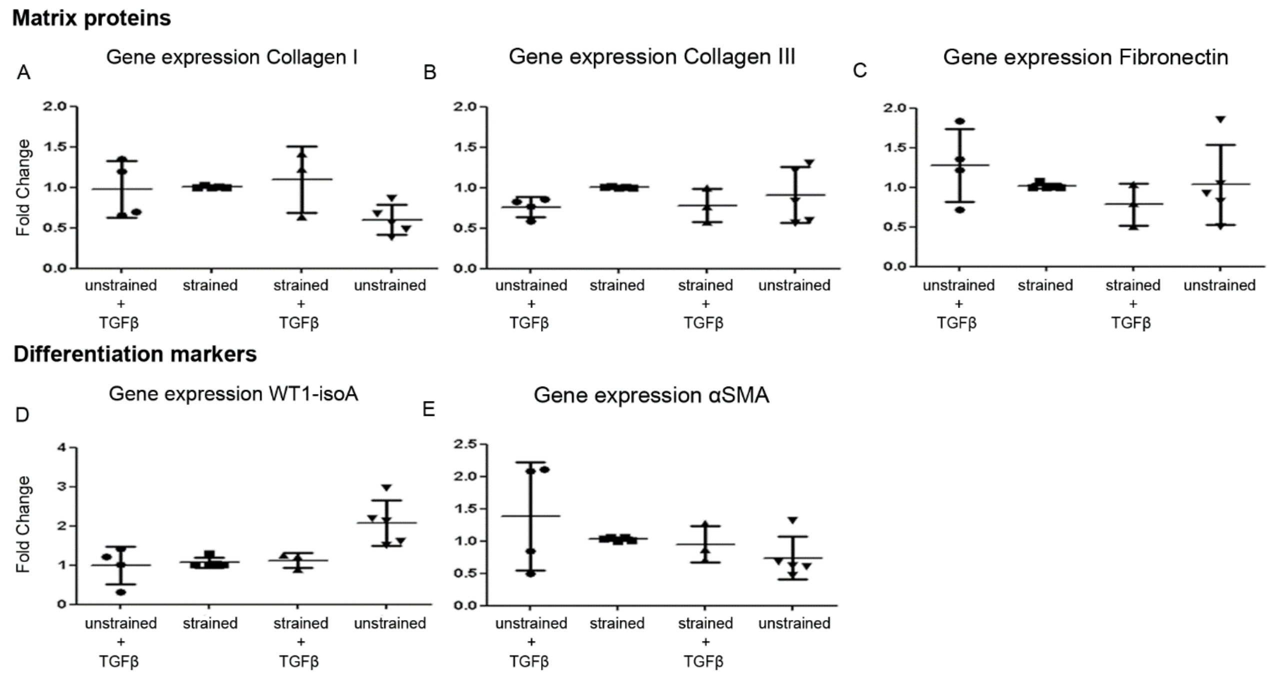

Figure S4. Cyclic strain and TGFβ have no additive effects on the matrix biosynthesis and differentiation potential of hEPDCs. Unstrained cells stimulated with TGFβ (0.5ng/ml), strained hEPDCs and exposure to strain simultaneously with TGFβ (0.5ng/ml) showed no changes in mRNA expression for matrix proteins collagen I (**A**), collagen III (**B**) and fibronectin (**C**). The process of differentiation of hEPDCs was also not altered with exposure to TGFβ under unstrained and strained conditions compared to hEPDCs under only cyclic strain conditions as shown by the expression of *WT1* (**D**) and *αSMA* (**E**). All data is represented compared to the strained samples.
